# *N*-Benzoimidazole/Oxadiazole Hybrid Universal Electron Acceptors for Highly Efficient Exciplex-Type Thermally Activated Delayed Fluorescence OLEDs

**DOI:** 10.3389/fchem.2019.00187

**Published:** 2019-04-03

**Authors:** Wenbo Yuan, Hannan Yang, Mucan Zhang, Die Hu, Ning Sun, Youtian Tao

**Affiliations:** ^1^Key Lab for Flexible Electronics and Institute of Advanced Materials (IAM), Nanjing Tech University, Nanjing, China; ^2^Department of Physics, Yunnan University, Kunming, China

**Keywords:** electron acceptor, electron donor, exciplex, oxadiazole, thermally activated delayed fluorescence, OLEDs

## Abstract

Recently, donor/acceptor type exciplex have attracted considerable interests due to the low driving voltages and small singlet-triplet bandgaps for efficient reverse intersystem crossing to achieve 100% excitons for high efficiency thermally activated delayed fluorescence (TADF) OLEDs. Herein, two *N*-linked benzoimidazole/oxadiazole hybrid electron acceptors were designed and synthesized through simple catalyst-free C-N coupling reaction. 24*i*PBIOXD and *i*TPBIOXD exhibited deep-blue emission with peak at 421 and 459 nm in solution, 397 and 419 nm at film state, respectively. The HOMO/LUMO energy levels were −6.14/−2.80 for 24*i*PBIOXD and −6.17/−2.95 eV for *i*TPBIOXD. Both compounds could form exciplex with conventional electron donors such as TAPC, TCTA, and mCP. It is found that the electroluminescent performance for exciplex-type OLEDs as well as the delayed lifetime was dependent with the driving force of both HOMO and LUMO energy offsets on exciplex formation. The delayed lifetime from 579 to 2,045 ns was achieved at driving forces close to or larger than 1 eV. Two TAPC based devices possessing large HOMO/LUMO offsets of 1.09–1.34 eV exhibited the best EL performance, with maximum external quantum efficiency (EQE) of 9.3% for 24*i*PBIOXD and 7.0% for *i*TPBIOXD acceptor. The TCTA containing exciplex demonstrated moderate energy offsets (0.88–1.03 eV) and EL efficiency (~4%), while mCP systems showed the poorest EL performance (EQE <1%) and shortest delayed lifetime of <100 ns due to inadequate driving force of 0.47–0.75 eV for efficient exciplex formation.

## Introduction

Organic light-emitting diodes (OLEDs) have been developed rapidly in recent years since the pioneer work on low-voltage fluorescence electroluminescence by Tang in 1987 (Tang and VanSlyke, 1987; Ma et al., 1998; Gong et al., 2010; Park et al., 2013; Zhang et al., 2016). According to spin statistics, the ratio for singlet and triplet excitons recombined from electrogenerated holes and electrons is 1:3 (Baldo et al., 1999; Segal et al., 2003). Thus, the first generation of traditional fluorescent OLEDs which solely harvest singlet excitons only shows 25% of maximum internal quantum efficiency (IQE) (Wen et al., 2005). On the other hand, the second generation of phosphorescent OLEDs (PHOLEDs) based on heavy metal complexes and third generation of thermally activated delayed fluorescence (TADF) OLEDs could both reach 100% IQE in theory by utilizing all singlet and triplet excitons through intersystem crossing (ISC) and reverse inter-system crossing (RISC), respectively (Baldo et al., 1998; Adachi et al., 2001; Su et al., 2008; Lo et al., 2009; Goushi et al., 2012; Uoyama et al., 2012; Zhang and Forrest, 2012; Li et al., 2016; Cao et al., 2017; Huang et al., 2018; Wu Q. et al., 2018). However, to avoid consuming noble metals and achieving reliable true-blue light, TADF OLEDs based on low-cost pure organic emitters have attracted increasing interests as an alternative mechanism to PHOLEDs. TADF emission is realized by an up-conversion process from lower energy triplet states to slightly higher energy singlet states by endothermic reverse inter-system crossing process (Li et al., 2016; Cao et al., 2017; Huang et al., 2018; Wu Q. et al., 2018). Therefore, a small singlet-triplet energy bandgap (Δ*E*_ST_) is required for TADF emitters.

It is reported that the small Δ*E*_ST_ could be attained in (i) intramolecular charge transfer featured single molecule with twisted donor-acceptor structured for effective spatial isolation between the highest occupied molecular orbital (HOMO) and lowest unoccupied molecular orbital (LUMO) on the relevant hole and electron transporting moieties, and (ii) bimolecular-exciplex which contains an electron-donor material mixed with an electron-acceptor material through intermolecular charge transfer characteristics (Cai and Su, 2018; Liu et al., 2018; Sarma and Wong, 2018). High external quantum efficiency (EQE) of 20% for red, 29% for orange, 38% for green, and 37% for light-blue TADF OLEDs have been achieved in single-molecule TADF emitters (Lin et al., 2016; Chen et al., 2018; Wu T.-L. et al., 2018; Zeng et al., 2018). However, the development of bimolecular TADF lags far behind. Most exciplex-type TADF OLEDs showed maximum EQE close to 10% (Jankus et al., 2014; Liu et al., 2015a,b; Oh et al., 2015; Zhang L. et al., 2015; Hung et al., 2016, 2017; Jeon et al., 2016), with only one example approaching to 18% (Liu et al., 2016).

In electron donor/acceptor formed exciplex systems, compared with commercially available various electron-donor materials, such as 4,4′,4″-tris[3-methylphenyl(phenyl)amino]-triphenylamine (*m*-MTDATA), *N,N*′-bis(1-naphthyl)-*N,N*′-diphenyl-[1,1′-biphenyl]-4,4′-diamine (NPB), 4,4′-(cyclohexane-1,1-diyl)bis(*N*-phenyl-N-*p*-tolylaniline) (TAPC), 4,4′,4″-tris(N-carbazolyl) triphenylamine (TCTA), 4,4′-bis(*N*-carbazolyl)-1,1′-biphenyl (CBP), and *N,N*′-dicarbazolyl-3,5-benzene (mCP) etc., the types of efficient and low-cost electron-acceptor materials are scarce (Goushi and Adachi, 2012; Goushi et al., 2012; Sun et al., 2014; Lee et al., 2015; Liu et al., 2015b). Thus, the exploration of electron accepting materials is essential for constructing exciplex systems with outstanding optoelectronic performance. Therefore, in this work, we designed and synthesized two new electron-acceptors of 2-(2,4-bis(2-phenyl-1*H*-benzo[*d*]imidazol-1-yl)phenyl)-5-phenyl-1,3,4-oxadiazole (24*i*PBIOXD), and 2-phenyl-5-(2,4,6-tris(2-phenyl-1*H*-benzo[*d*]imidazol-1-yl)phenyl)-1,3,4-oxadiazole (*i*TPBIOXD) through a simple one-step catalyst-free aromatic nucleophilic substitution reaction. The electron-withdrawing oxadiazole (OXD) unit has been extensively applied in donor-acceptor type bipolar transport host materials, single molecule intramolecular charge transfer type TADF emitters as well as electron transport materials (Tao et al., 2011; Mondal et al., 2013; Olivier et al., 2017; Cooper et al., 2018; Yao et al., 2018; Zhang et al., 2018). By combining OXD building block with our previously reported isomeric *N*-linkaged benzoimidazole (Hu et al., 2017), both 24*i*PBIOXD, and *i*TPBIOXD exhibited deep HOMO level of ~-6.15 eV, facilitating the exciplex formation with general electron donor materials of TAPC, TCTA, and mCP due to the compatible HOMO and LUMO energy levels between donor and acceptor materials. The gradient energy offsets ranging from 0.47 to 1.34 eV correlated well with the delayed lifetime and EL efficiencies in exciplex type TADF OLEDs. The TAPC:24*i*PBIOXD exciplex with the largest HOMO/LUMO offsets exhibited the best EL performance, with maximum EQE of 9.3% for green TADF OLEDs.

## Results and Discussion

### Synthesis and Characterization

**Scheme 1:**
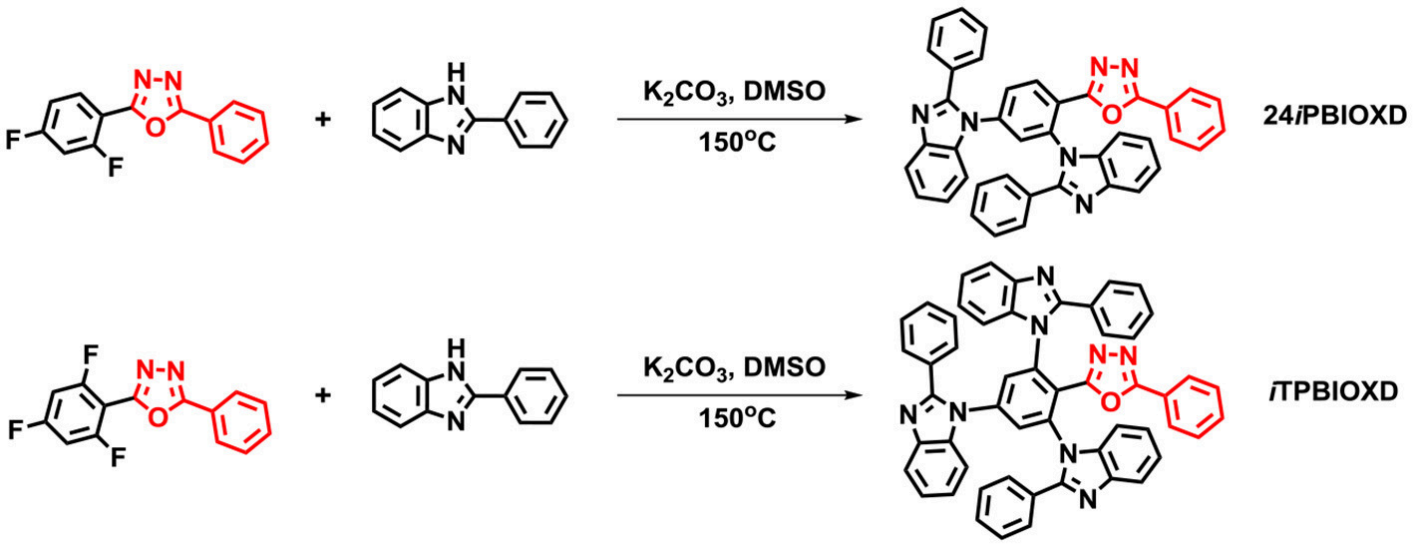
Synthesis of compounds 24*i*TPBIOXD and *i*TPBIOXD.

**Figure 1:**
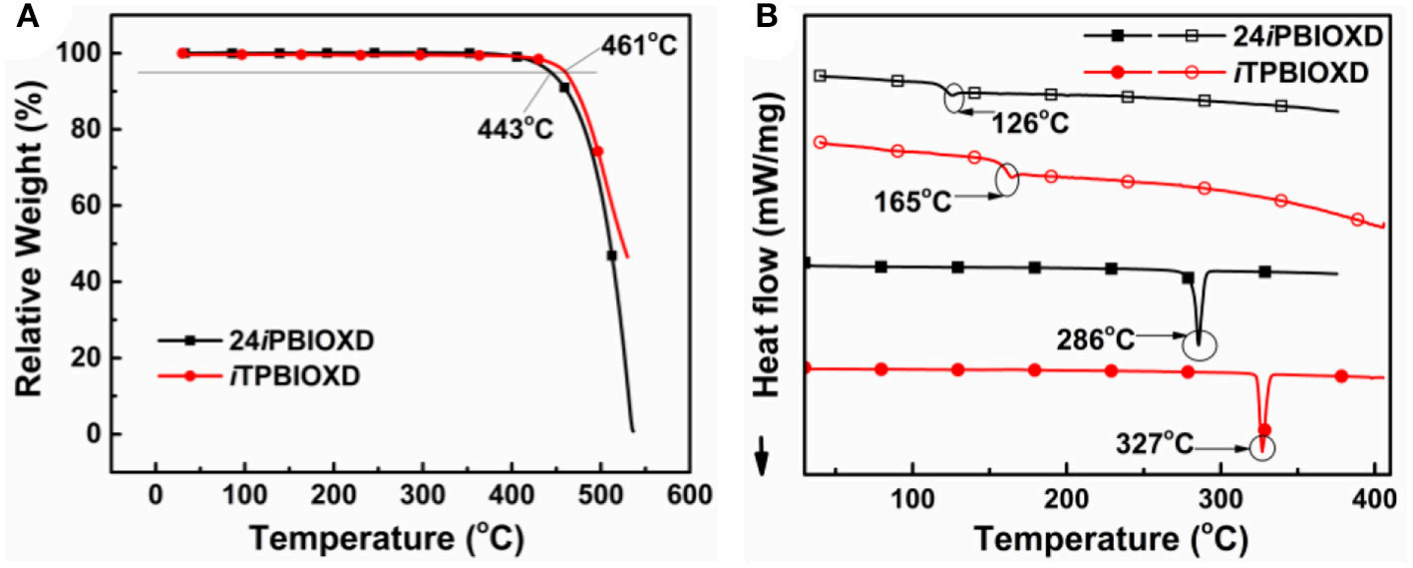
**(A)** Thermogravimetric analysis (TGA) and **(B)** differential scanning calorimetry (DSC) (solid symbols represent for first heating scan and open symbols for second heating scan) curves for 24*i*PBIOXD and *i*TPBIOXD.

Scheme 1 shows the synthetic routes and molecular structures of 24*i*PBIOXD and *i*TPBIOXD. The two compounds could be facilely synthesized by a simple one-step catalyst free C-N coupling reaction. This nucleophilic substitution reaction was carried out in DMSO solvent with K_2_CO_3_ base at high yields over 80% by using di/tri-fluorine substituted oxadiazole derivatives as electrophiles and 2-phenyl-1*H*-benzo[*d*]imidazole as nucleophiles. The considerably high yields and environmentally eco-friendly conditions demonstrated the superiority than common metal-catalyzed Ullman reactions (Son et al., 2008; Liu et al., 2011; Volz et al., 2013). In addition, the directly connection of the isomeric *N*-linked benzoimidazole to the central phenyl ring avoided the complicated multistep ring-closing synthetic process for the normal *C*-linked benzoimidazole in traditional electron transport material of 2,2,2-(1,3,5-phenylene)-tris(1-phenyl-1*H*-benzoimidazole) (TPBI) or its derivatives. The chemical structures of the new compounds were fully characterized by ^1^H NMR, ^13^C NMR, mass spectrometry (MALDI-TOF) and element analysis (Figure S1). The good thermal stability of the two compounds was confirmed by thermogravimetric analysis (TGA) and differential scanning calorimetry (DSC) (Figure 1). The decomposition temperatures (*T*_d_, corresponding to a 5% weight loss) from TGA curves for 24*i*PBIOXD and *i*TPBIOXD were determined at 443 and 461°C, respectively. Additionally, the melting point (*T*_m_) of *i*TPBIOXD was observed at 327°C, which was much higher than 286°C of 24*i*PBIOXD. The glass transition temperature (*T*_g_) of both materials can be detected from the second heating cycles from DSC, with values of 126°C for 24*i*PBIOXD and 165°C for *i*TPBIOXD, indicating their reasonable thermal stability.

### Photophysical Properties

**Figure 2:**
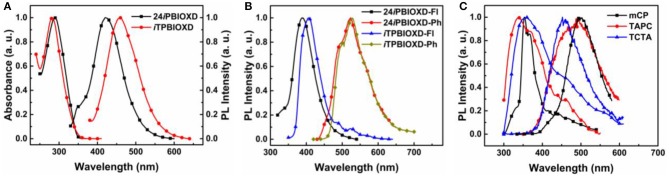
**(A)** Normalized UV-Vis absorption and PL spectra for 24*i*TPBIOXD and *i*TPBIOXD in CH_2_Cl_2_ solution, **(B)** 77 K fluorescence (Fl) and phosphorescence (Ph) spectra for 24*i*TPBIOXD and *i*TPBIOXD in neat film, **(C)** 77 K fluorescence (Fl) and phosphorescence (Ph) spectra for three donor materials in neat film.

**Figure 3:**
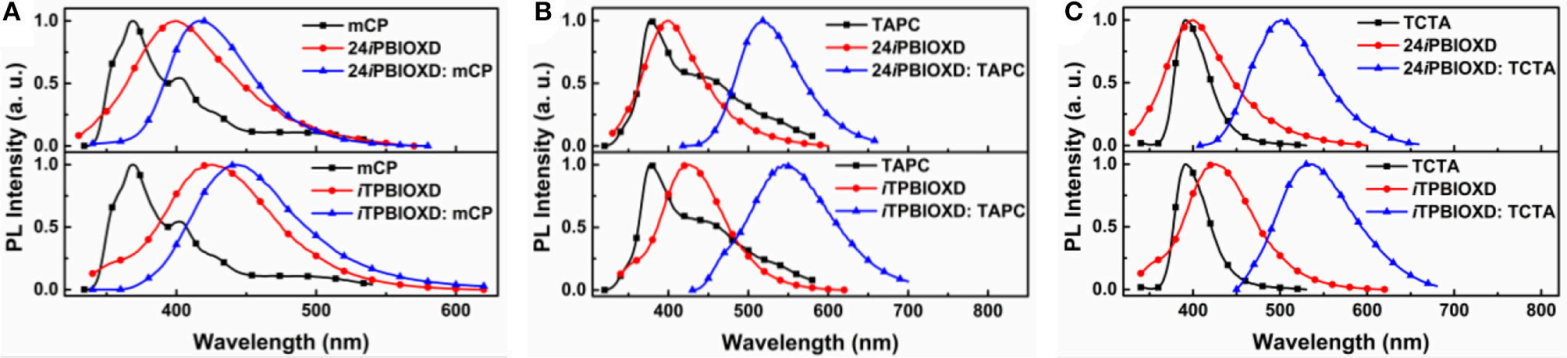
PL spectra of **(A)** mCP, **(B)** TAPC, **(C)** TCTA with different electron acceptors and the relevant exciplexes in film.

**Figure 4:**
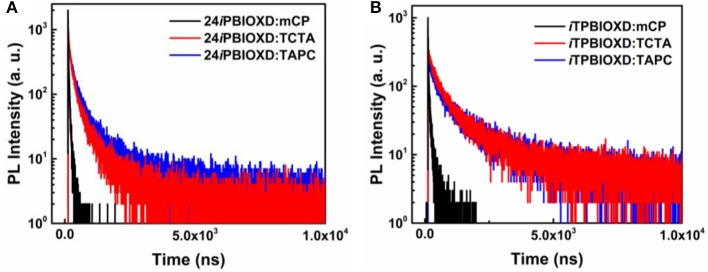
Transient decay curves of **(A)** 24iPBIOXD and **(B)** iTPBIOXD-based exciplexes at film state.

The room temperature UV-Vis absorption and photoluminescence (PL) spectra of 24*i*PBIOXD and *i*TPBIOXD in CH_2_Cl_2_ solution are shown in Figure 2A. Both compounds exhibited an intense absorption with peaks at 289 and 283 nm in solution, 297 and 288 nm in film, respectively, which can be ascribed to the π*-*π^*^ transition of molecules. The optical bandgap (*E*_g_) was calculated to be 3.34 eV for 24*i*PBIOXD and 3.22 eV for *i*TPBIOXD, according to the film-state absorption edge. On the other hand, 24*i*PBIOXD and *i*TPBIOXD showed unimodal photoluminescence peaking at 421 and 459 nm in solution, whereas significantly blue-shift to 397 and 419 nm in film state (Table 1). By analyzing the highest-energy vibronic sub-band of low-temperature fluorescence and phosphorescence spectrum (Figure 2B), the singlet (*E*_S_) and triplet (*E*_T_) energy levels could be determined to be 3.31/2.55 and 3.18/2.53 eV for 24*i*PBIOXD and *i*TPBIOXD, respectively. In addition, the *E*_S_/*E*_T_ energy levels of three hole-transport electron donor materials were also calculated to be 3.54/2.95 eV for mCP, 3.79/2.82 eV for TAPC, and 3.66/2.84 eV for TCTA (Figure 2C). The PL spectra for the neat film of electron donors such as mCP, TAPC, and TCTA, the two new electron-acceptors of 24*i*PBIOXD and *i*TPBIOXD as well as their corresponding mixtures in a 1:1 weight ratio were investigated. As shown in Figure 3 and Figure S2, all blended films showed bathochromic shifted PL spectra compared with the emission of neat 24*i*PBIOXD/*i*TPBIOXD and the corresponding donor-material, indicating the successful formation of exciplex (Zhang T. et al., 2015). In addition, it is found that exciplex based on 24*i*PBIOXD acceptors all exhibited about 20–30 nm blue-shifted emission than *i*TPBIOXD based exciplex systems. The exciplex emission color could be tuned from deep-blue of mCP:24*i*PBIOXD with peak at 419 nm to light-blue of TCTA:24*i*PBIOXD (501 nm) and further to green of TAPC:24*i*PBIOXD (518 nm). Besides, transient photoluminescence (PL) measurements were carried out for all six exciplexes (Figure 4). The exciplexes comprising TAPC or TCTA donor all possessed significantly longer delayed decay lifetime, with values of 579 ns for TCTA:24*i*PBIOXD, 1,907 ns for TCTA:*i*TPBIOXD, 1,520 and 2,045 ns for TAPC:24*i*PBIOXD, TAPC:*i*TPBIOXD exciplex, respectively. However, the mCP:24*i*PBIOXD and mCP:*i*TPBIOXD exciplex systems displayed greatly shorter delayed decay lifetime of only 42 and 72 ns (Table 1). Besides, the temperature dependent PL transients for the representative TAPC:24*i*PBIOXD and TCTA:*i*TPBIOXD exciplexes (Figure S3) both demonstrated a more significant decay from 100 to 300 K at the longer lifetime range, suggesting the potential existence of endothermic reverse inter-system crossing. It is expected the obvious variations on delayed decay time for different exciplexes may demonstrate some relationships with the device efficiency in exciplex-TADF OLEDs.

**Table 1:** Physical properties of compounds 24*i*PBIOXD and *i*TPBIOXD.

**Compounds**	**λ_**abs**_/λ_em_^a^ [nm]**	**λ_**abs**_/λ_em_^b^ [nm]**	***E*_**S**_/*E*_T_^c^ [eV]**	***E*_g_^d^ [eV]**	**HOMO/LUMO^e^ [eV]**	***T*_**g**_/*T*_**m**_/*T*_d_^f^ [°C]**	***λ***_**CT**_ **(nm)/*****E***_**CT**_ **(eV)/*****τ***_**d**_ **(ns)/Δ*****E*** **(eV)**^**g**^		
							**mCP**	**TCTA**	**TAPC**
24*i*PBIOXD	289/421	297/397	3.31/2.55	3.34	−6.14 (−5.75)/−2.80 (−2.09)	126/286/443	419/2.96/42	501/2.48/579	518/2.39/1520
							0.47/0.6	0.95/0.88	1.09/1.19
*i*TPBIOXD	283/459	288/419	3.18/2.53	3.22	−6.17 (−5.95)/−2.95(−2.05)	165/327/461	443/2.80/72	531/2.36/1907	544/2.28/2045
							0.5/0.75	0.98/1.03	1.12/1.34

a *Measured in CH_2_Cl_2_ solution at room temperature*.

b *Measured in film*.

c *Singlet energy and triplet energy was calculated from low temperature (77 K) fluorescence spectra and phosphorescence spectrum*.

d *Optical bandgap (E_g_) calculated from the absorption edge of film state UV-Vis spectra*.

e *LUMO measured from the onset of reduction curves from CV and HOMO calculated from the difference between LUMO and E_g_, values in parentheses from DFT calculations*.

f *Glass transition temperature/melting point/decomposition temperature*.

g *Emission maxima, charge transfer state energy, delayed decay lifetime and HOMO/LUMO energy offsets for various exciplexes*.

### Theoretical Calculations and Electrochemical Properties

**Figure 5:**
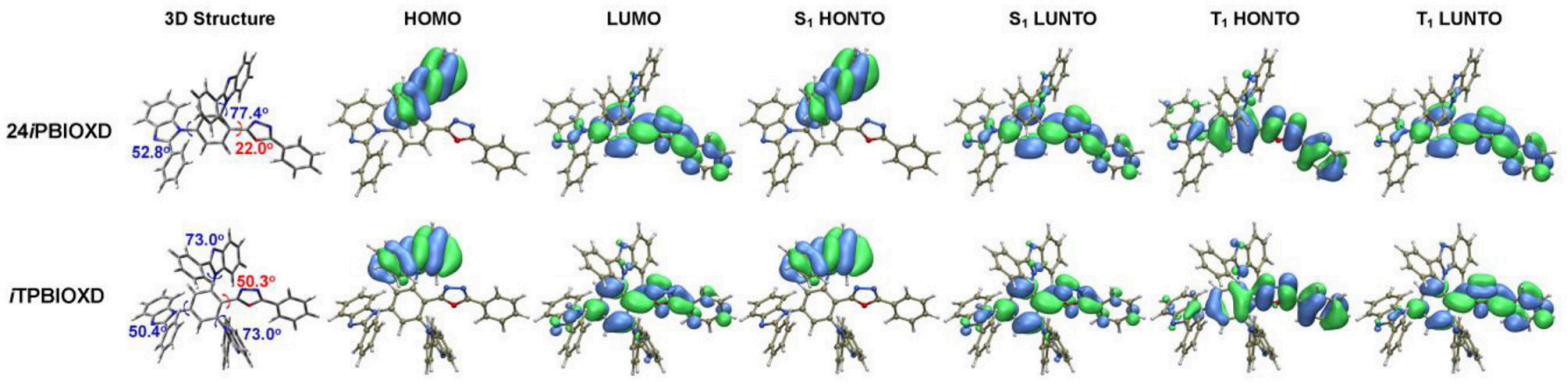
Optimized 3D structures, spatial distributions of ground state HOMO and LUMO, singlet (S_1_), and triplet excited state (T_1_) HONTO and LUNTO for compounds 24*i*PBIOXD and *i*TPBIOXD.

In order to gain insights into the frontier molecular orbital and excited states level distribution of 24*i*PBIOXD and *i*TPBIOXD, density functional theory (DFT) calculation was conducted at the B3LYP level (Francl et al., 1982; Becke, 1988; Lee et al., 1988). From the optimized geometry shown in Figure 5, the dihedral angles between the central phenyl and oxadiazole ring were 22.0 and 50.3° for 24*i*PBIOXD and *i*TPBIOXD, respectively, the values between the benzoimidazoles and the central phenyl rings ranged from 50.4 to 77.4°, indicating a twisted structure for both compounds. Furthermore, in the ground state, the highest occupied molecular orbital (HOMO) were almost completely located on one of the ortho-positioned phenylbenzoimidazole units, indicating the electron-donating characteristics of *N*-linked phenylbenzoimidazole, which was quite different from the *C*-isomerized phenylbenzoimidazole containing TPBI (Hu et al., 2017). And the lowest unoccupied molecular orbital (LUMO) were mainly localized on 2,5-diphenyl-1,3,4-oxadiazole, along with mildly distribution over the penta-heterocyclic imidazoles, suggesting the weak electron-withdrawing property to gently participate electron-transport for the imidazoles. Similar distribution can be observed in the highest occupied natural transition orbital (HONTO) and the lowest unoccupied natural transition orbital (LUNTO) at singlet excited state. It should be noted that the HONTO distribution at triplet excited state was completely different from S_0_ and S_1_ for both compounds, which was mainly delocalized through the 2,5-diphenyl-1,3,4-oxadiazole skeleton, similar with the LUNTO distribution.

**Figure 6:**
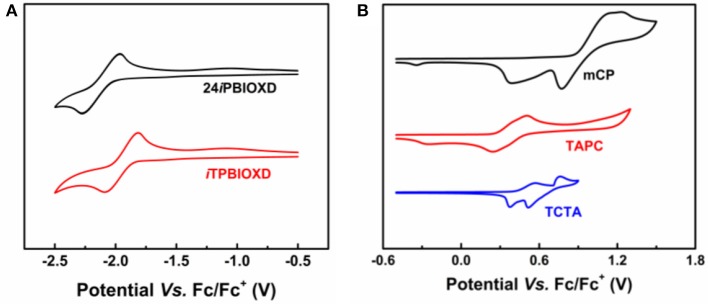
Cyclic voltammograms of **(A)** 24*i*PBIOXD and *i*TPBIOXD in THF solution for reduction scan; **(B)** conventional electron donors (mCP, TAPC, and TCTA) in CH_2_Cl_2_ solution for oxidation scan.

**Figure 7:**
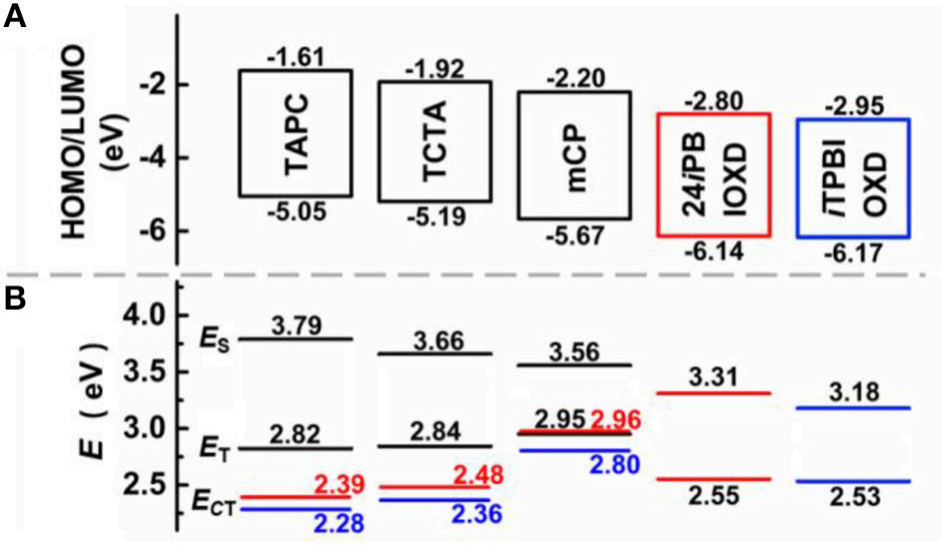
**(A)** HOMO, LUMO energy level diagrams and **(B)** singlet and triplet excited-state energies for 24*i*PBIOXD, *i*TPBIOXD, and various donor materials, as well as the charge transfer state energy (*E*_CT_) for the corresponding exciplex systems, red for 24*i*PBIOXD, and blue for *i*TPBIOXD.

The electrochemical features were measured by cyclic voltammetry (CV) (Figure 6). Both compounds exhibited reversible reduction whereas undetectable oxidation behavior. The LUMO energy levels calculated from the onset of reduction curves for 24*i*PBIOXD and *i*TPBIOXD were measured to be −2.80 and −2.95 eV, while the HOMO energy levels calculated from the different between the LUMO and optical bandgaps (*E*_g_) were evaluated to be −6.14 and −6.17 eV, respectively. The values were in good agreement with the theoretical calculation. Besides, the energy levels for electron donor materials were also measured, with HOMO estimated from the onset of electro-oxidation curves and LUMO calculated from HOMO and optical bandgaps. The HOMO/LUMO energy level values for mCP, TAPC, and TCTA were −5.67/−2.20, −5.05/−1.61, and −5.19/−1.92 eV, respectively. The deep HOMO and LUMO for the two new electron acceptors of 24*i*PBIOXD and *i*TPBIOXD, provided sufficient driving forces on HOMO/LUMO energy offsets for the exciplex formation (Figure 6A). As shown in Figure 7A, the HOMO energy level offsets between the electron donor of TAPC, TCTA, or mCP and the electron acceptors of 24*i*PBIOXD/*i*TPBIOXD were calculated to be 1.09/1.12, 0.95/0.98, or 0.47/0.5 eV, and the corresponding LUMO offsets were 1.19/1.34, 0.88/1.03, or 0.6/0.75 eV, respectively. It is noted in both acceptor systems, the TAPC donor based exciplex presented the highest driving force, followed by TCTA, while the mCP donor demonstrated the lowest HOMO/LUMO offsets.

### Electroluminescence Properties

**Figure 8:**
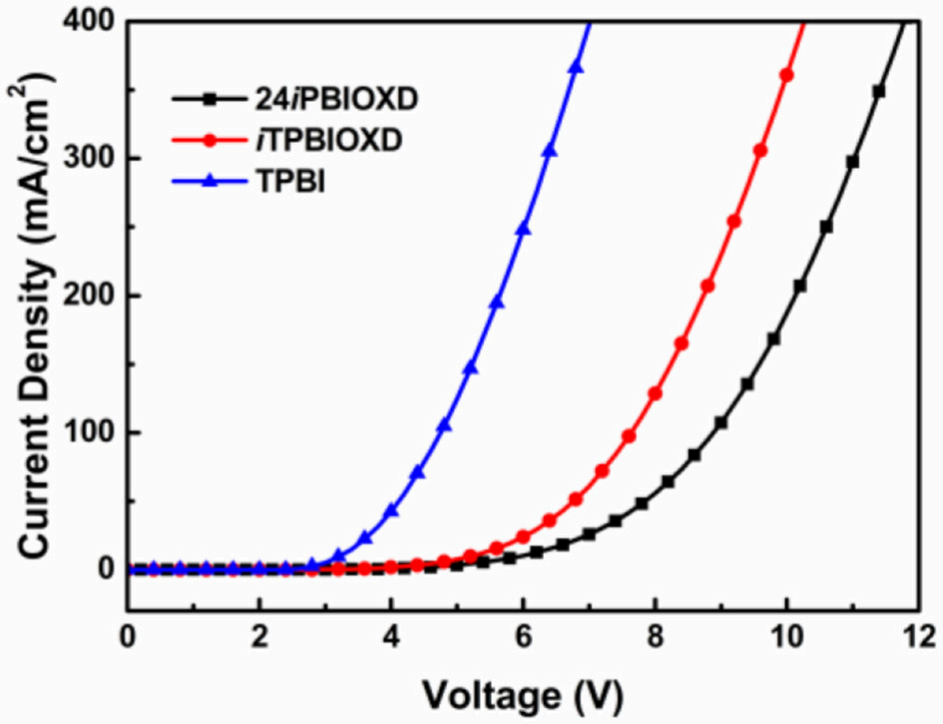
*J-V* characteristic of nominal single-electron-only devices based on compounds 24*i*PBIOXD, *i*TPBIOXD, and TPBI [device structures: ITO/EML (50 nm)/LiF (1 nm)/Al (150 nm)].

To investigate the charge transport properties of the two new *N*-linked isomeric benzoimidazole containing electron acceptors, single carrier electron-only device was prepared to find out the electron inject and transport properties of 24*i*PBIOXD and *i*TPBIOXD. The device structure was ITO/24*i*PBIOXD, *i*TPBIOXD, or TPBI (50 nm)/LiF (1 nm)/Al (150 nm), where the commercial electron transport material of 2,2,2-(1,3,5-phenylene)-tris(1-phenyl-1*H*-benzoimidazole) (TPBI) with *C*-linkage in benzoimidazole was selected for comparison. As shown in Figure 8, at the same operating voltage, TPBI based device exhibited the highest current density among all the three devices. Since the LUMO energy of TPBI (2.7–2.9 eV) (Bian et al., 2018; Jou et al., 2018) was almost the same as 24*i*PBIOXD and *i*TPBIOXD, which manifested their similar injection barrier for efficient electron injection. Therefore, the significantly higher current for TPBI indicated better electron transporting property than 24*i*PBIOXD and *i*TPBIOXD. On the other hand, the current density in *i*TPBIOXD device was slightly higher than 24*i*PBIOXD, as depicted in Figure 8, the LUMO level of *i*TPBIOXD was 0.15 eV lower than 24*i*PBIOXD, therefore a mildly efficient electron-injection could be attained in *i*TPBIOXD device due to its lower injection barriers. Thus, the electron-transport performance for both electron acceptors may be comparable.

To conduct a comprehensive comparison on the EL performance for the exciplex-TADF OLEDs among diverse electron-donor and acceptor systems, a series of vacuum deposited devices A-F were fabricated. Due to the highest HOMO level of TAPC for efficient hole-injection, the device configuration for TAPC-based OLEDs was ITO/MoO_3_ (1 nm)/TAPC:24*i*PBIOXD or *i*TPBIOXD (1:1, 70 nm)/LiF (1 nm)/Al (100 nm). To reduce the hole-injection barrier, TCTA-based device was constructed by ITO/MoO_3_ (1 nm)/TAPC (40 nm)/TCTA:24*i*PBIOXD or *i*TPBIOXD (1:1, 30 nm)/TmPyPB (40 nm)/LiF (1 nm)/Al (100 nm), while a further 10 nm TCTA thin film was inserted between the TAPC layer and emissive layer (EML) in mCP-based devices. Among them, MoO_3_ and LiF were used as hole- and electron-injection materials, respectively; TAPC and 1,3,5-tri[(3-pyridyl)-phen-3-yl] benzene (TmPyPB) were functionalized as hole- and electron-transport materials, respectively, an extra TCTA layer was aimed to promote the hole-injection and block the electrons.

**Figure 9:**
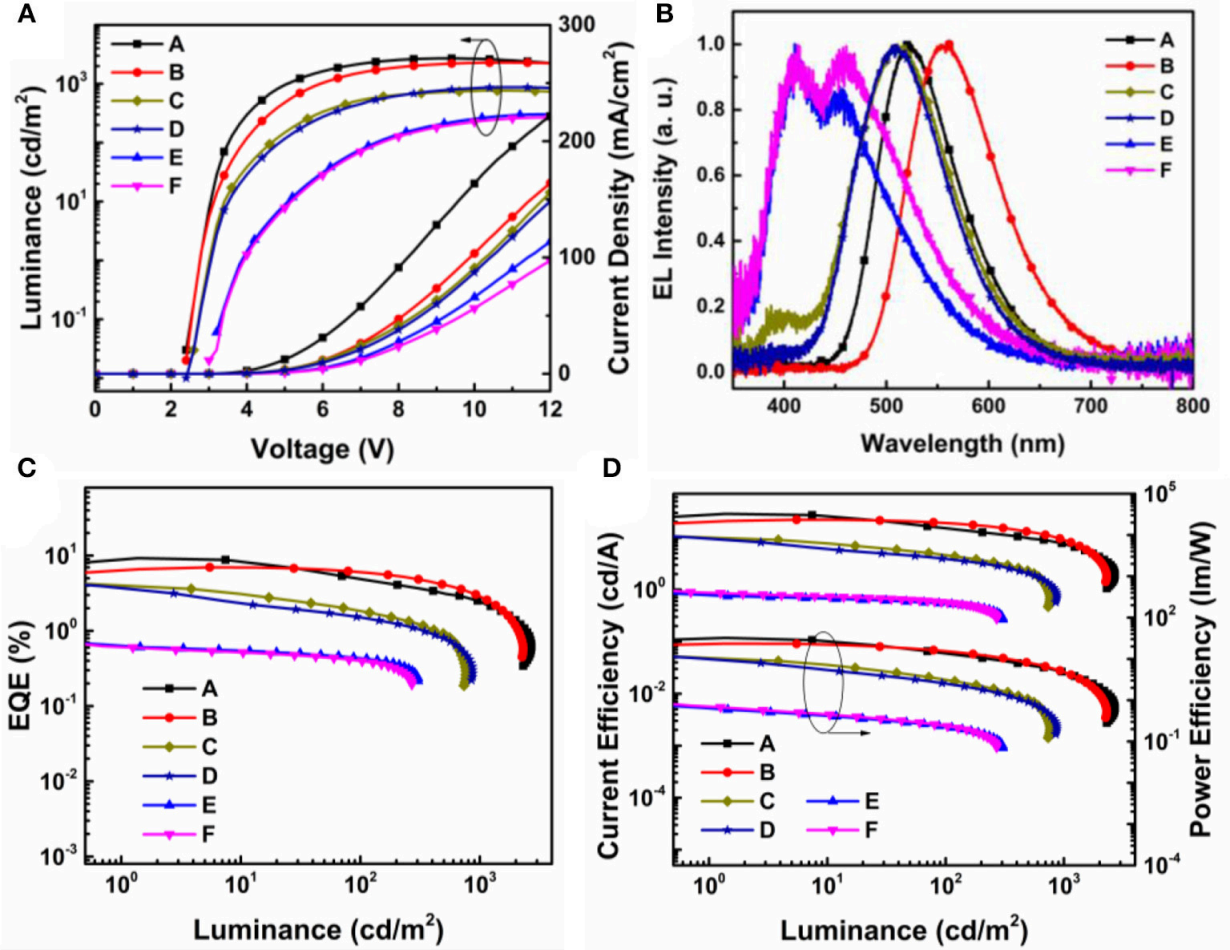
**(A)** *L-V-J* characteristics; **(B)** normalized electroluminescent (EL) spectra; **(C)** external quantum efficiency (EQE) vs. luminance curves; **(D)** current efficiency and power efficiency vs. luminance curves of device A–F.

The current density-voltage-luminance (*J-V-L*), electroluminescence (EL) spectra, together with the current and power efficiency, external quantum efficiency vs. luminance curves are shown in Figure 9. The device fabrication details are stated in Supporting Information. According to the key EL data listed in Table 2, the turn-on voltage for TAPC, TCTA, and mCP containing devices was gradiently increased from 2.8, 3.0 to 3.8 eV. The as high as 0.48 eV hole-injection barrier between TCTA and mCP lead to the highest operating voltage in devices E and F. The EL performance trend was in consistent with the values of HOMO/LUMO energy offsets, and TAPC-analog bearing the highest driving forces for convenient exciplex formation demonstrated the best highest EL efficiency. The best performance was attained from device A with TAPC:24*i*PBIOXD exciplex, corresponding to a maximum current efficiency (CE), power efficiency (PE), and external quantum efficiency (EQE) of 28.8 cd/A, 32.3 lm/W, and 9.3%. And the TAPC:*i*TPBIOXD based device B demonstrated slightly poorer EL performance with maximum current efficiency, power efficiency and external quantum efficiency of 22.1 cd/A, 23.4 l m/W, and 7.0%, respectively. Device C and D based on TCTA electron donor showed comparable EL efficiency, with maximum EQE of 4.0 and 3.9% for 24*i*PBIOXD and *i*TPBIOXD electron acceptors, respectively. The EL performance for mCP device was rather poor, with maximum EQE of <1% in both device E and F. As depicted in Figure 9B, devices A and B with TAPC donor depicted smooth exciplex-TADF emission, with EL peak at 519 and 556 nm, respectively, which is in agreement with the relevant PL spectra. Commission Internationale de L'Eclairage (CIE) values for device A and B was measured at (0.31, 0.55) and (0.43, 0.54), corresponding to green and yellow emission, respectively. The TCTA based device C and D both exhibited blueish-green emission, with a gentle shoulder peak at around 400 nm for 24*i*PBIOXD. The two mCP-based devices displayed blue emission with CIE x, y each at ~0.20. However, the EL spectra of device E and F revealed bimodal emission showing comparable intensity for the two peaks. It is hypothesized that the inadequate HOMO and LUMO energy offsets (< ~1 eV) for TCTA:24*i*PBIOXD, mCP:24*i*PBIOXD, and mCP:*i*TPBIOXD, resulted in the unexpected shorter wavelength EL emission peak, which was ascribed to pure mCP emission (Chiu and Lee, 2012; Shahalizad et al., 2017). In addition, since the *E*_CT_ of TAPC and TCTA based exciplexes were lower than the triplet energy of both donor and acceptor materials, which was beneficial to restrict triplet excitons in the exciplex states for the efficient RISC. However, *E*_CT_ of 2.8–2.96 eV (Figure 7B) for mCP based exciplex was significantly higher than the triplet energy (~2.55 eV) of the two electron acceptors, which provided a potential way for energy leakage from exciplex states to the T_1_ excited state of 24*i*PBIOXD and *i*TPBIOXD. Thus, devices based on mCP donors demonstrated the lowest EL efficiency and inadequate TADF emission.

**Table 2:** Electroluminescence characteristics for the devices.

**Device**	**Emitting layer**	***V*_on_^a^ (V)**	**η_c_^b^ (cd/A)**	**η_p_^c^ (lm/W)**	**η_ext_^d^ (%)**	**CIE (x,y)**
A	TAPC: 24*i*PBIOXD	2.8	28.8	32.3	9.3	(0.31, 0.55)
B	TAPC: *i*TPBIOXD	2.8	22.1	23.4	7.0	(0.43, 0.54)
C	TCTA: 24*i*PBIOXD	3.0	10.1	10.6	4.0	(0.26, 0.43)
D	TCTA: *i*TPBIOXD	3.0	10.6	10.2	3.9	(0.25, 0.44)
E	mCP: 24*i*PBIOXD	3.8	0.8	0.66	0.65	(0.19, 0.20)
F	mCP: *i*TPBIOXD	3.8	0.9	0.74	0.63	(0.20, 0.23)

a *Turn-on voltage at 1 cd/m^2^*.

b *Maximum current efficiency*.

c *Maximum power efficiency*.

d *Maximum external quantum efficiency*.

## Conclusion

In summary, we have designed and synthesized two universal *N*-linked benzoimidazole/oxadiazole hybrid electron acceptors through a simple nucleophilic substitution reaction. Diverse deep-blue to yellow emissive exciplex could be formed between various conventional donor materials and the two acceptors due to their deep HOMO levels of ~6.15 eV. The HOMO and LUMO energy level offsets which were also named as the driving forces for exciplex formation were gradiently increased from 0.47 to 1.12 and 0.6 to 1.34 eV in mCP, TCTA, to TAPC based exciplexes. We have found that both HOMO and LUMO offsets ≥1 eV was required to form efficient and stable intermolecular charge transfer exciplex. When the driving forces were as low as 0.47–0.75 eV, which is far <1 eV, the two mCP based exciplex demonstrated considerably short delayed component lifetime, with values of only 42 ad 72 ns for 24*i*PBIOXD and *i*TPBIOXD acceptors, respectively. Additionally, the exciplex-type device EQE was lower than 1%. When the driving forces were slightly lower or approaching 1 eV, the two TCTA exciplexes displayed moderate EL efficiency of about 4%. And the best EL performance was achieved in TAPC containing exciplex-type TADF OLEDs, with relatively low turn-on voltage of 2.8 V, maximum efficiency of 28.8 cd/A CE, 32.3 lm/W PE, and 9.3% for 24*i*PBIOXD acceptor and 22.1 cd/A CE, 23.4 lm/W and 7.0% for *i*TPBIOXD acceptor. Our results provide guidance on the exploration of efficient exciplex type TADF OLEDs.

## Author Contributions

WY, MZ, and DH designed and synthesized the materials. WY and MZ did most of the experimental work and data analyses. OLED device fabrication and electroluminescent performance studies were carried out by HY and NS. YT had the idea, led the project. WY and YT prepared the manuscript. All authors contributed to the manuscript preparation.

### Conflict of Interest Statement

The authors declare that the research was conducted in the absence of any commercial or financial relationships that could be construed as a potential conflict of interest.
