# Supplementary material for: N-Benzoimidazole/Oxadiazole Hybrid Universal Electron Acceptors for Highly Efficient Exciplex-Type Thermally Activated Delayed Fluorescence OLEDs
Source: Front Chem. 2019 Apr 3;7:187. doi: 10.3389/fchem.2019.00187 (PMC6457096; doi:10.3389/fchem.2019.00187)
Supplement: Supplementary file 1 [file Data_Sheet_1.doc]

Supplementary Material

# Experimental Section

## General Information

^1^H NMR spectra and ^13^C NMR were recorded on a Bruker DMX-500 spectrometer in deuterated dimethylsulfoxide using tetramethylsilane (TMS; *δ* = 0 ppm) as an internal standard. Mass spectra were recorded using a Bruker Autoflex matrix assisted laser desorption/ionization time-of-flight (MALDI-TOF). Elemental analyses (EA) of C, H, N, and S were performed on a Vario EL III microanalyzer. Absorption spectra: Ultraviolet-visible (UV–Vis) absorption spectra of solution in chlorobenzene and thin film on a quartz substrate were measured using Shimadzu UV-2500 recording spectrophotometer, and photoluminescence (PL) spectra were recorded using a Hitachi F-4600 fluorescence spectrophotometer. The transient lifetime was carried out using Edinburgh FLS-980 Instruments in the film state. Thermal analysis: Thermal gravimetric analysis (TGA) was undertaken with a METTLER TOLEDO TGA2 instrument. The thermal stability of the samples was determined by measuring their weight loss at a heating rate of 10^o^C min^-1^ from 25 to 500^o^C using 3 mg sample under a nitrogen atmosphere. Differential scanning calorimetry (DSC) was performed on a NETZSCH DSC 200 PC unit within the temperature range of 50 to 300 ^o^C, heating at a rate of 10 ^o^C min^-1^ under N2 atmosphere. Cyclic voltametry (CV): The electrochemical cyclic voltammetry was conducted on a CHI voltammetric analyzer, in a 0.1 mol L^-1^ acetonitrile solution of tetrabutylammonium hexafluorophosphate (n-Bu_4_NPF_6_) at a potential scan rate of 100 mV s^-1^. The conventional three electrode configuration consists of a platinum working electrode, a platinum wire counter electrode, and an Ag/AgCl wire pseudo-reference electrode. The polymer sample was coated on the platinum sheet of working electrode. The reference electrode was checked versus ferrocenium-ferrocene (Fc^+^/Fc) as internal standard as recommended by IUPAC (the vacuum energy level: 24.8 eV). All the solutions were deaerated by bubbling nitrogen gas for a few minutes prior to the electrochemical measurements. HOMO energy levels were calculated from the equation of *E*_HOMO_ = - (E_onset_ (ox) + 4.8) eV, and LUMO energy levels were deduced from the optical band gap (*E*_g_) values and HOMO levels.

## Computation Details

The ground-state geometries were optimized with Gaussian 09 program package using the B3LYP (Becke three parameters hybrid functional with Lee-Yang-Perdew correlation) functional and 6-31G(d) basis set.^[1-3]^ The highest occupied and lowest unoccupied natural transition orbitals (HONTO and LUNTO) of T_1_ and S_1_ were both simulated at the wB97XD/6-311G(d,p) level. 3-D structure and molecular orbitals were visualized using Gauss view.

## Synthesis

All materials and solvents were purchased from Energy Chemical Co., Sigma Aldrich Co., Alfa Aesar without further purification.

Synthesis of 2-(2,4-bis(2-phenyl-1*H*-benzo[*d*]imidazol-1-yl)phenyl)-5-phenyl-1,3,4-oxadiazole (24*i*PBIOXD): A mixture of 2-(2,4-difluorophenyl)-5-phenyl-1,3,4-oxadiazole (0.5 g, 1.94 mmol), 2-phenyl-1*H*-benzo[*d*]imidazole (1.13 g, 5.81 mmol) and K_2_CO_3_ (2.14 g, 15.49 mmol) in dimethyl sulfoxide (DMSO) (15 mL) was stirred at 150^o^C for more than 12 h under an N_2_ atmosphere. After cooling to room temperature, the mixture was poured into water, filter, and then purified by column chromatography over silica gel with CH_2_Cl_2_/petroleum ether as the eluent to afford a white solid (Yield: 85%). ^1^H NMR (300 MHz, DMSO) δ = 8.40 (d, J=8.4, 1H), 8.12 (d, J=2.1, 1H), 7.94 (dd, J=8.4, 2.2, 1H), 7.89 – 7.82 (m, 2H), 7.67 – 7.47 (m, 11H), 7.44 – 7.31 (m, 6H), 7.30 – 7.17 (m, 3H), 7.00 – 6.93 (m, 1H). ^13^C NMR (75 MHz, DMSO) δ = 163.92, 160.69, 152.02, 142.75, 140.29, 136.77, 136.31, 135.35, 132.21, 131.91,128.40, 126.36, 123.12, 121.54, 119.55, 119.26, 110.37, 110.20. MALDI-TOF Mass (m/z): calcd for C_40_H_26_N_6_O: 606.22; found: 606.62. Anal. Calcd. For C_40_H_26_N_6_O: C 79.19, H 4.32, N 13.85%; found: C 79.61, H 4.12, N 13.86%.

Synthesis of 2-phenyl-5-(2,4,6-tris(2-phenyl-1*H*-benzo[*d*]imidazol-1-yl)phenyl)-1,3,4-oxadiazole (*i*TPBIOXD): A mixture of 2-phenyl-5-(2,4,6-trifluorophenyl)-1,3,4-oxadiazole (0.5 g, 1.81 mmol), 2-phenyl-1*H*-benzo[*d*]imidazole (1.58 g, 8.15 mmol) and K_2_CO_3_ (3.0 g, 21.72 mmol) in dimethyl sulfoxide (DMSO) (15 mL) was stirred at 150^o^C for more than 12 h under an N_2_ atmosphere. After cooling to room temperature, the mixture was poured into water, filter, and then purified by column chromatography over silica gel with CH_2_Cl_2_/petroleum ether as the eluent to afford a white solid (Yield: 85%). ^1^H NMR (300 MHz, DMSO) δ = 8.48 (s, 1H), 8.18 (s, 1H), 7.90 (d, J=7.6, 1H), 7.84 – 7.61 (m, 8H), 7.62 – 7.43 (m, 5H), 7.41 – 7.06 (m, 14H), 6.77 (dd, J=14.2, 7.5, 3H), 6.45 (s, 1H). ^13^C NMR (75 MHz, DMSO) δ = 163.89, 163.74, 157.02, 156.63, 152.41, 152.06, 151.78, 151.40, 142.90, 142.45, 142.40, 140.93, 137.14, 135.90, 132.36, 132.17, 128.84, 128.06, 126.03, 122.96, 121.68, 121.08, 119.74, 119.60, 119.43, 110.53, 110.05, 109.76. MALDI-TOF Mass (m/z): calcd for C_53_H_34_N_8_O: 798.29; found: 798.56. Anal. Calcd. For C_53_H_34_N_8_O: C 79.68, H 4.29, N 14.03%; found: C 79.27, H 4.15, N 13.83%.

## Device Fabrication and Measurements

The exciplex-type TADF OLEDs based on 24*i*TPBIOXD and *i*TPBIOXD were fabricated by vacuum deposition technology, using commercially available various electron-donor materials of 4,4′-(cyclohexane-1,1-diyl)bis(*N*-phenyl-N-*p*-tolylaniline) (TAPC), 4,4′,4″-tris(N-carbazolyl) triphenylamine (TCTA) and *N,N*′-dicarbazolyl-3,5-benzene (mCP) with the configuration of ITO/MoO_3_ (1 nm)/EML (1:1, 70 nm)/LiF (1 nm)/Al (100 nm). TCTA-based device was constructed by ITO/MoO_3_ (1 nm)/TAPC (40 nm)/TCTA:24*i*PBIOXD or *i*TPBIOXD (1:1, 30 nm)/TmPyPB (40 nm)/LiF (1 nm)/Al (100 nm), while a further 10 nm TCTA thin film was inserted in mCP-based devices. All layers were fabricated on pre-treated ITO substrates, which was cleaned in an ultrasonic bath with acetone and isopropanol solvents, and followed by oxygen plasma for 30 min. Firstly, 1 nm of MoO_3_ was deposited on ITO substrates as hole injection layer, alternatively followed by a 4,4′-cyclohexylidenebis[N,N-bis(4-methylphenyl)aniline] (TAPC) or a 4,4′,4″-tris(N-carbazolyl) triphenylamine (TCTA) layer for effectively transport and blocking. The exciplex emissive layers were then deposition on the films mentioned above. Finally, the electron transport layer was 1,3,5-tri(m-pyrid-3-ylphenyl)benzene (TmPyPB) for 40 nm, meanwhile, the 1 nm of LiF and hundred-nanometer of Al were considered as the cathode layers.

# Supplementary Figures and Tables


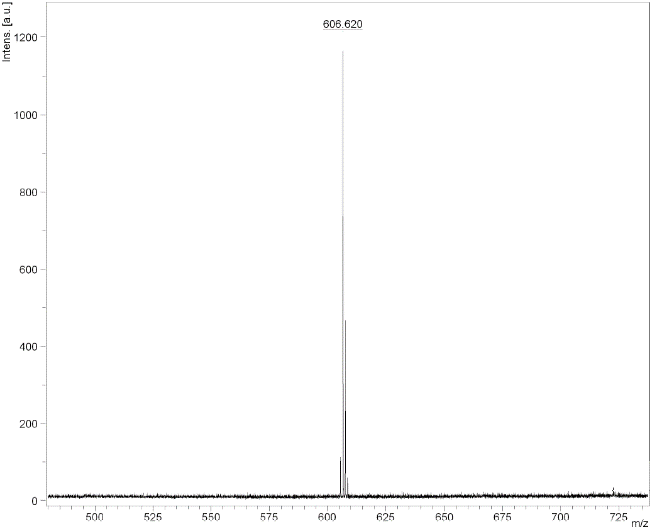

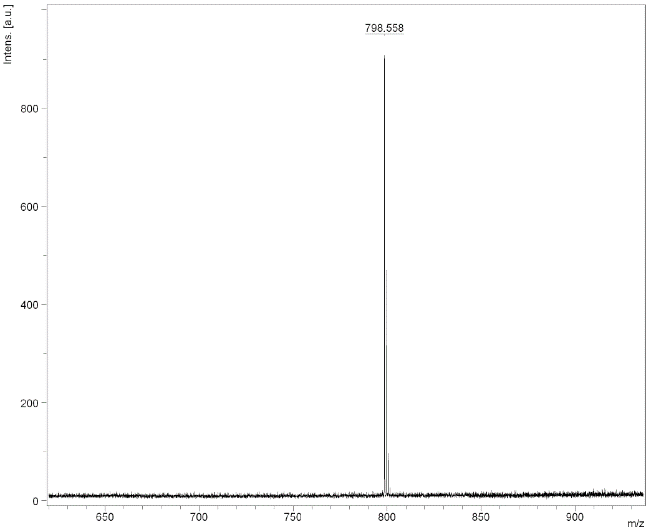


**Figure S1.** ^1^H NMR spectrum (up line), ^13^C NMR spectrum (middle line) and mass spectrometry (down line) of compounds 24*i*PBIOXD (left column) and *i*TPBIOXD (right column).

**Figure S2.** Normalized UV-Vis absorption and PL spectra of exciplexes in neat film state at room temperatu
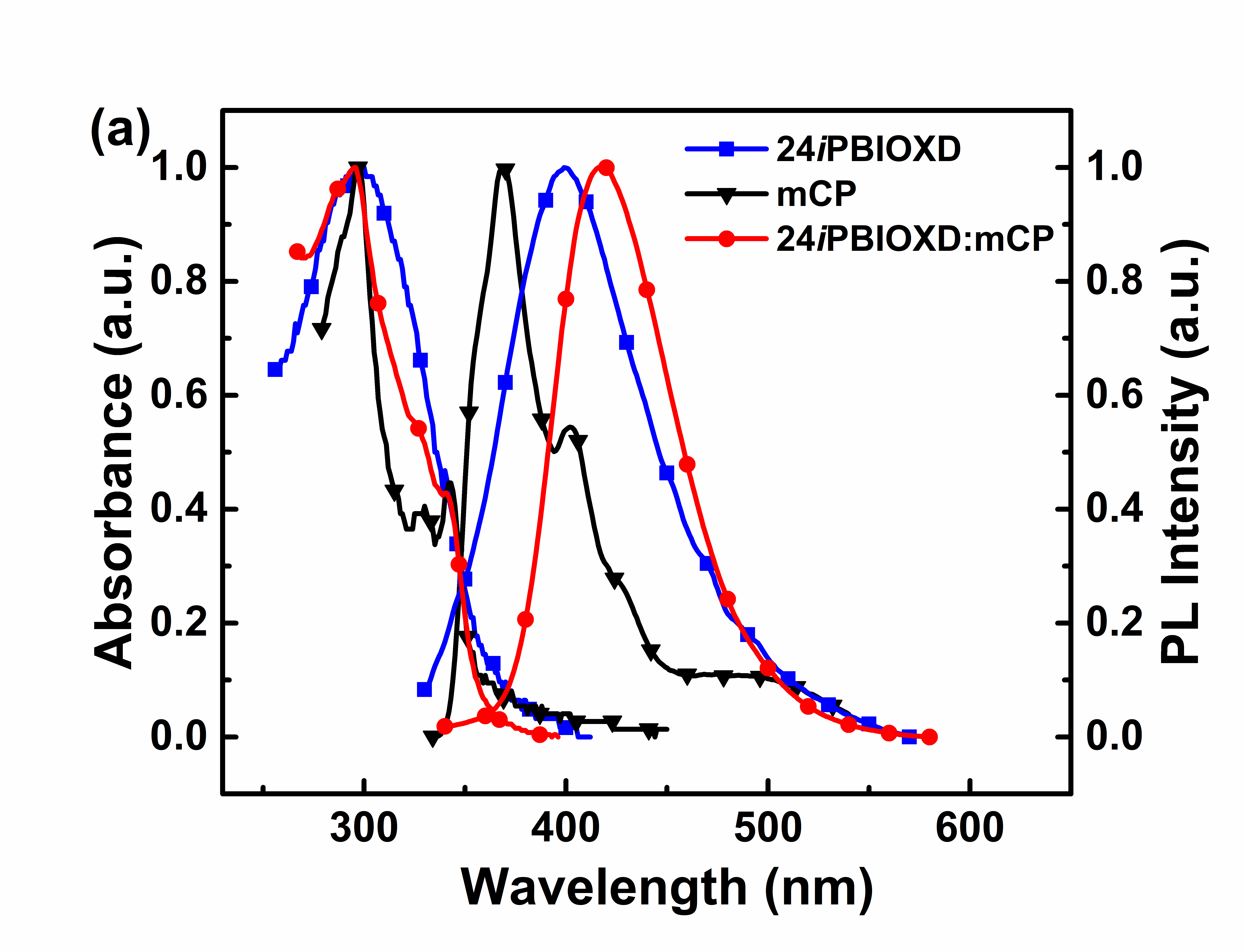

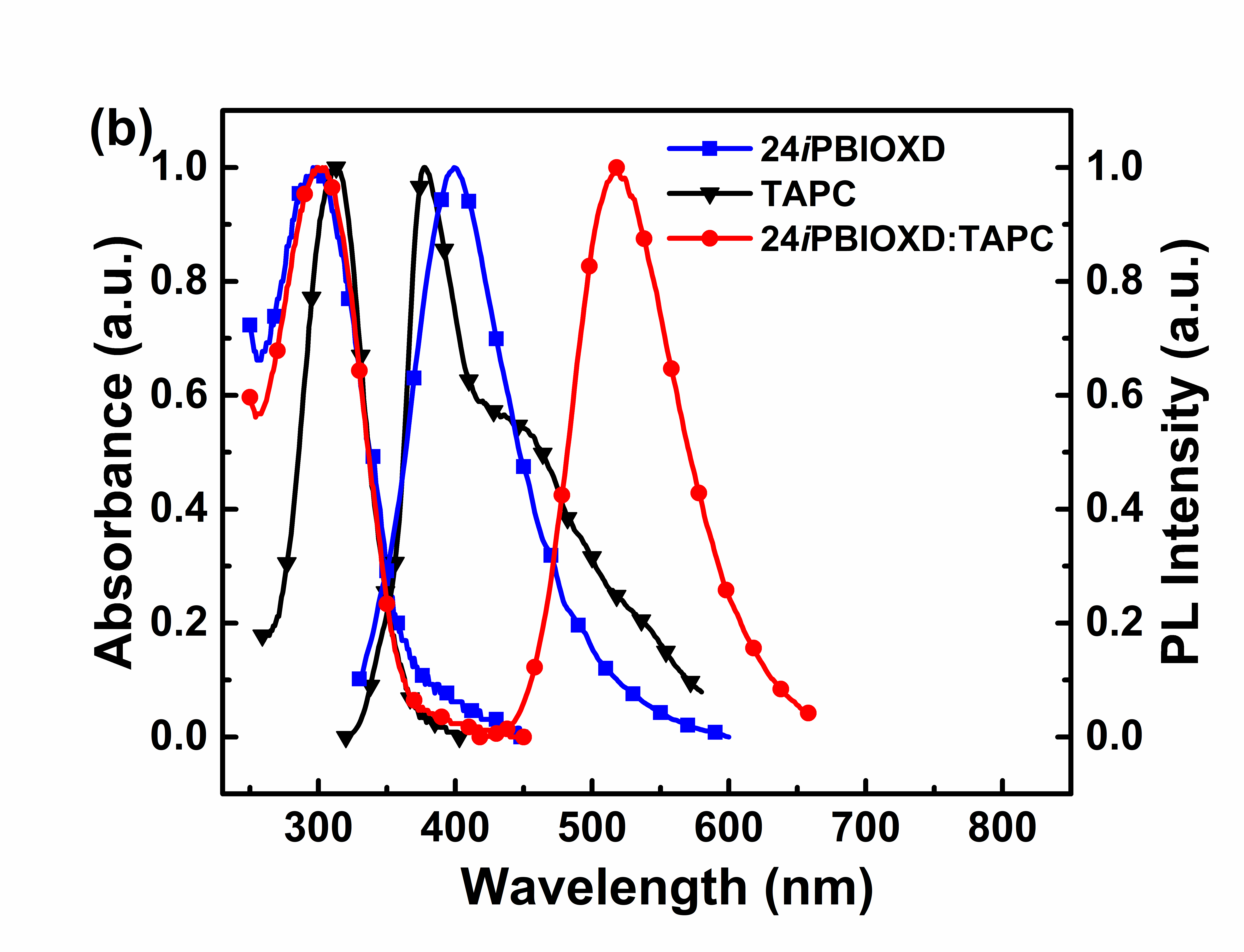

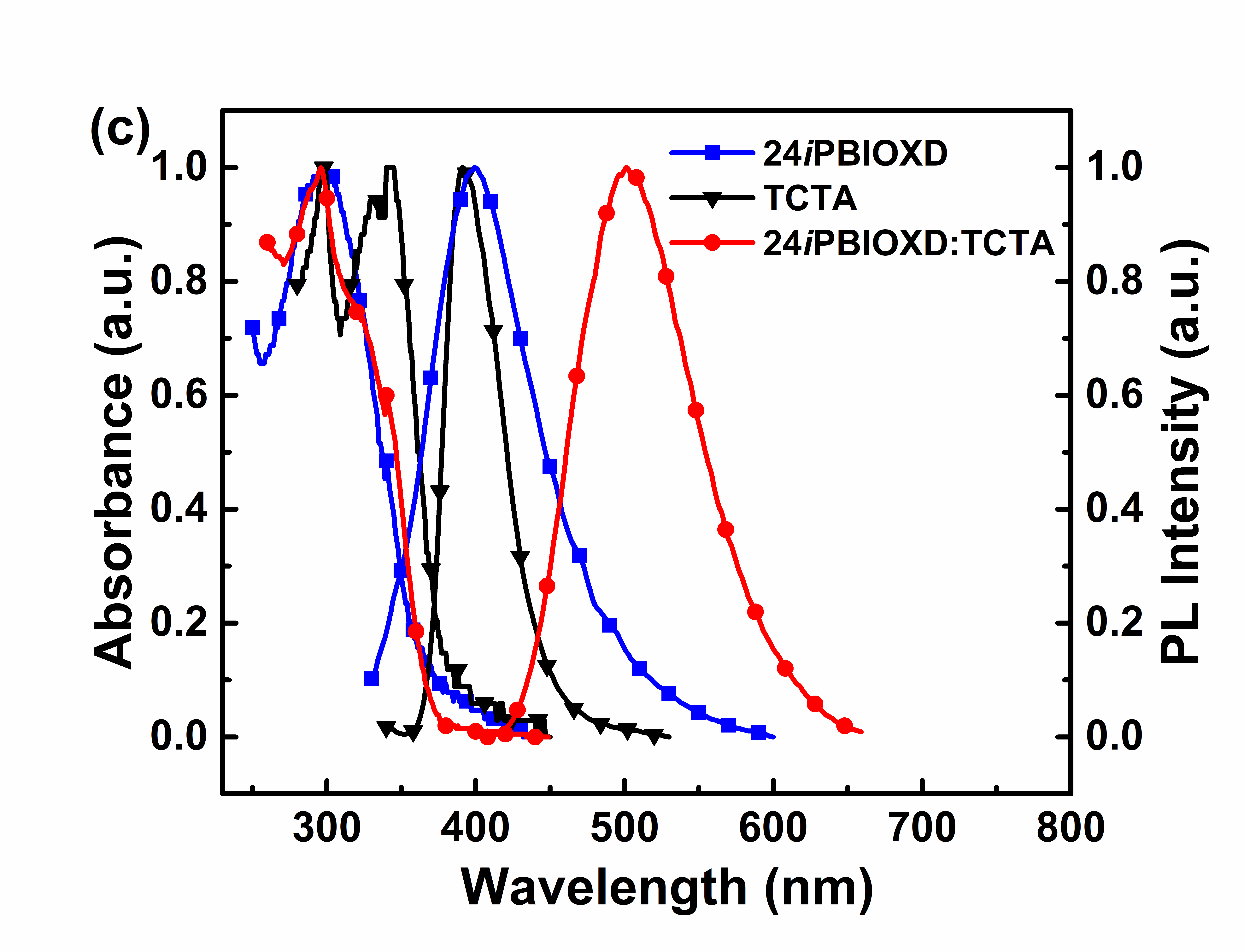

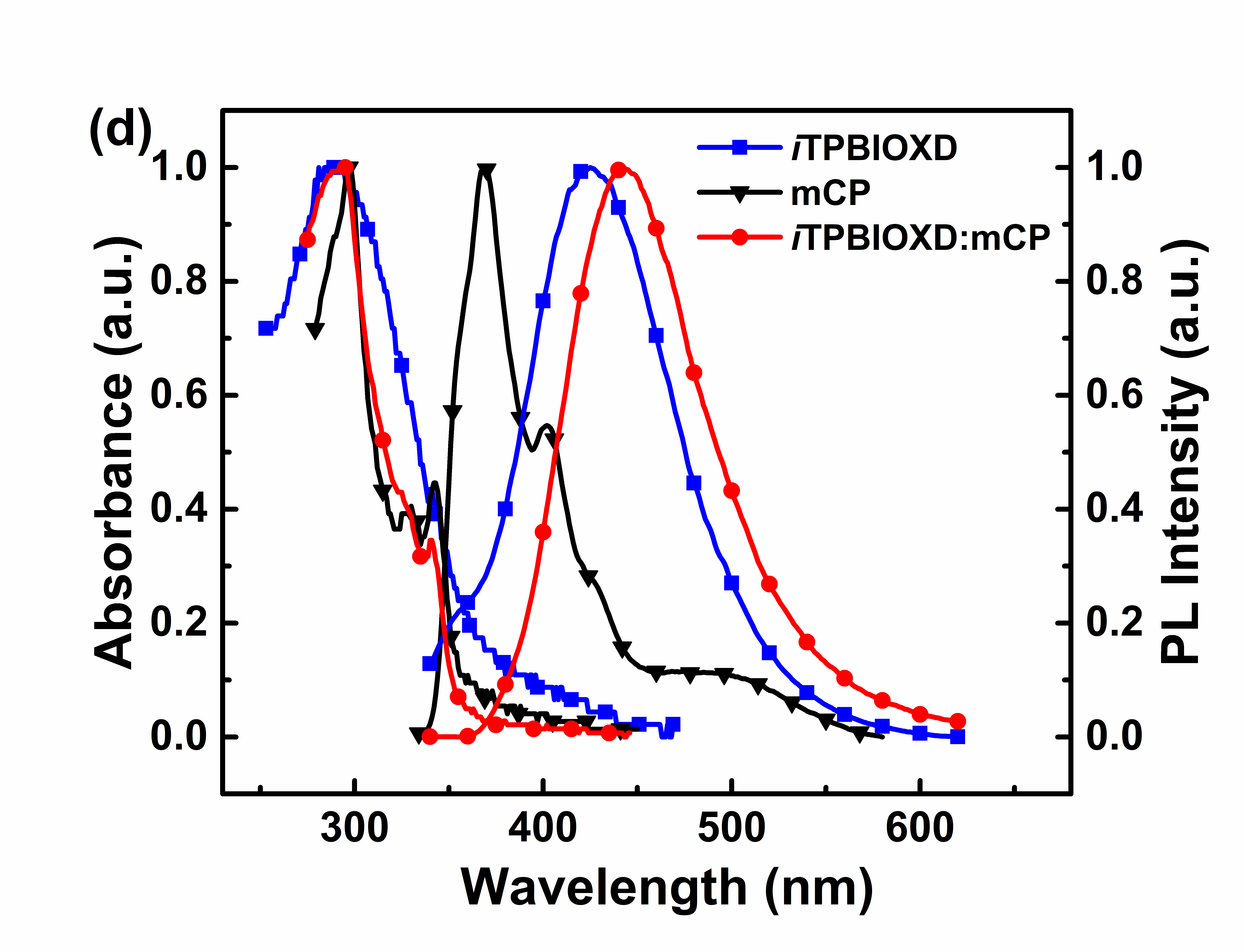

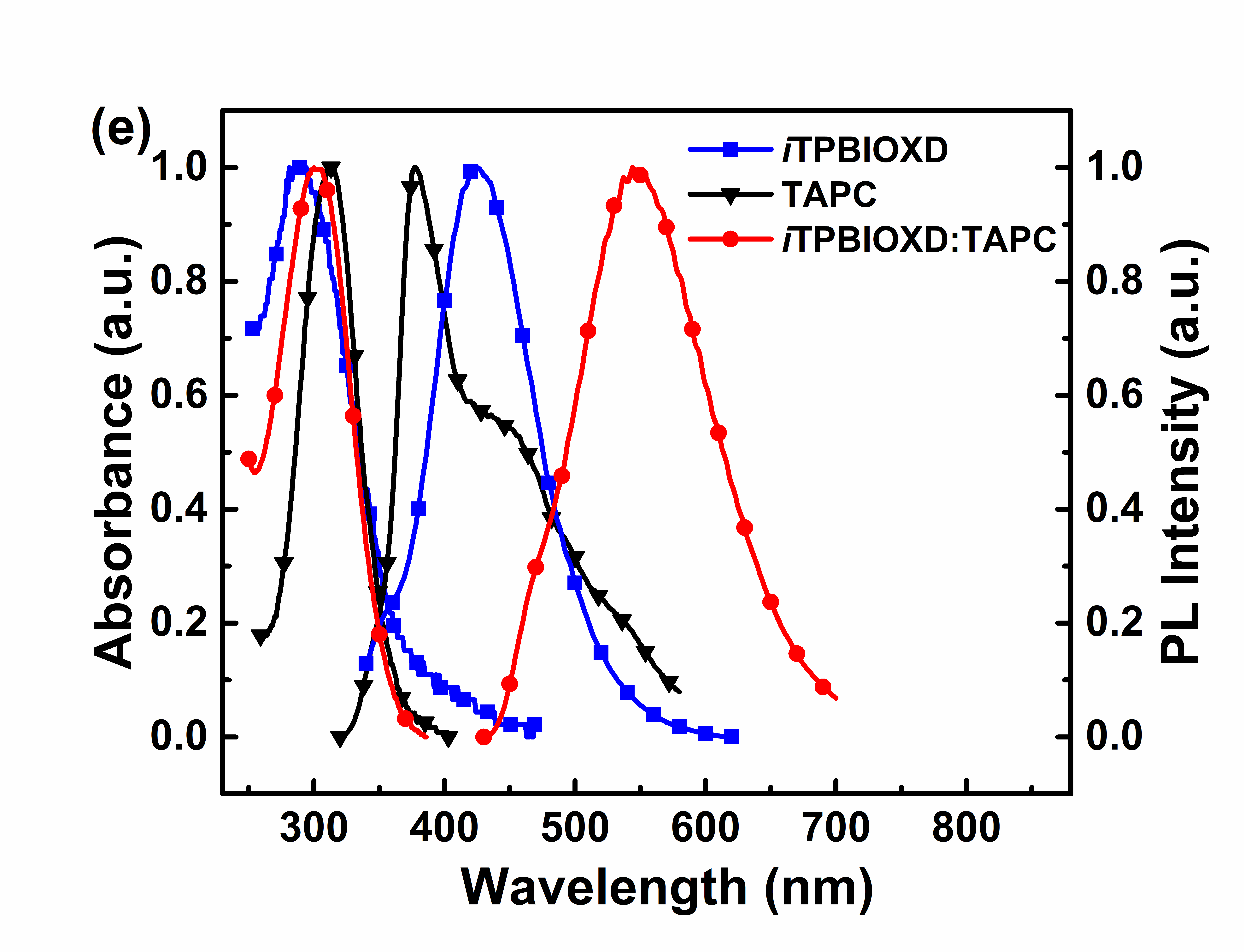

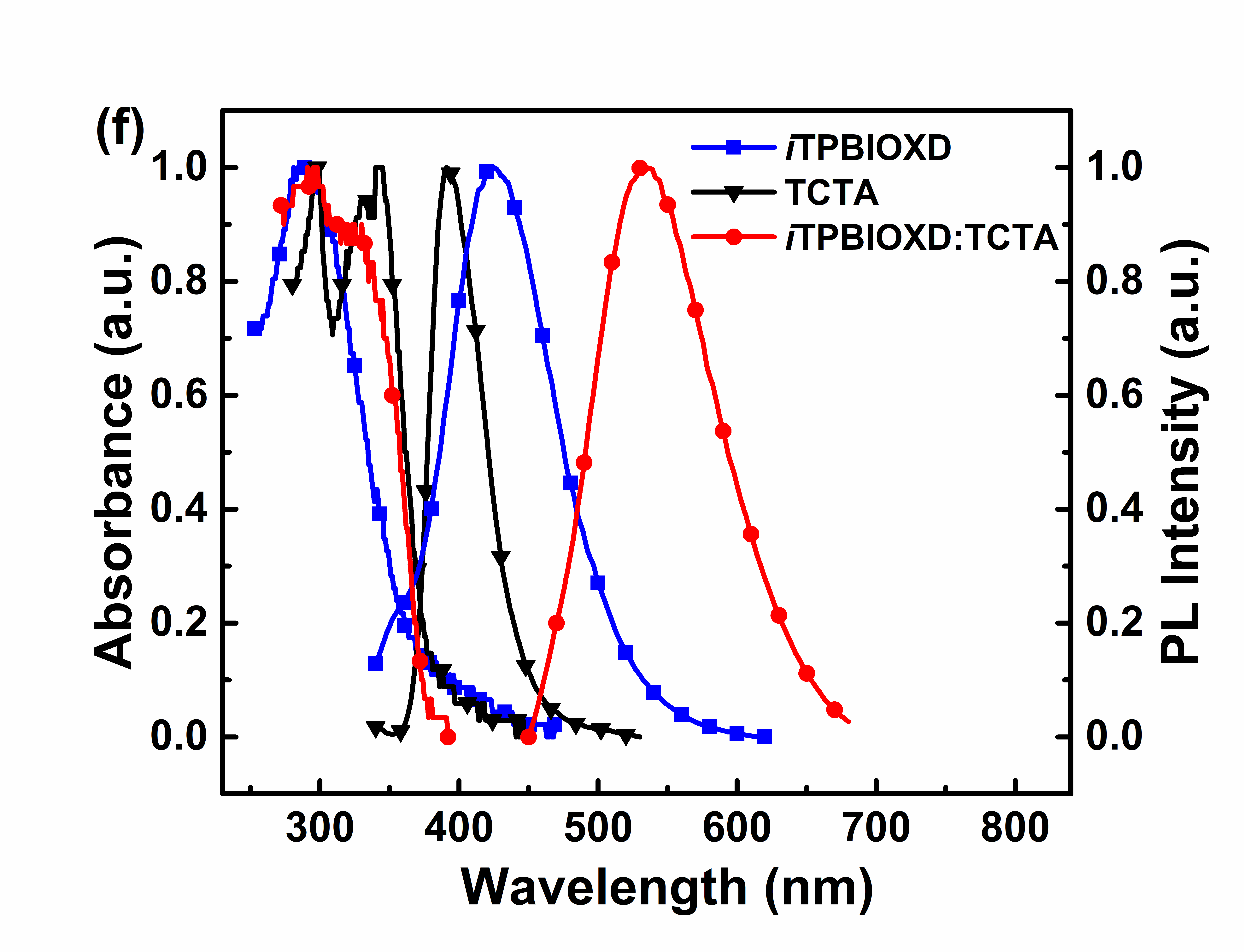
re.


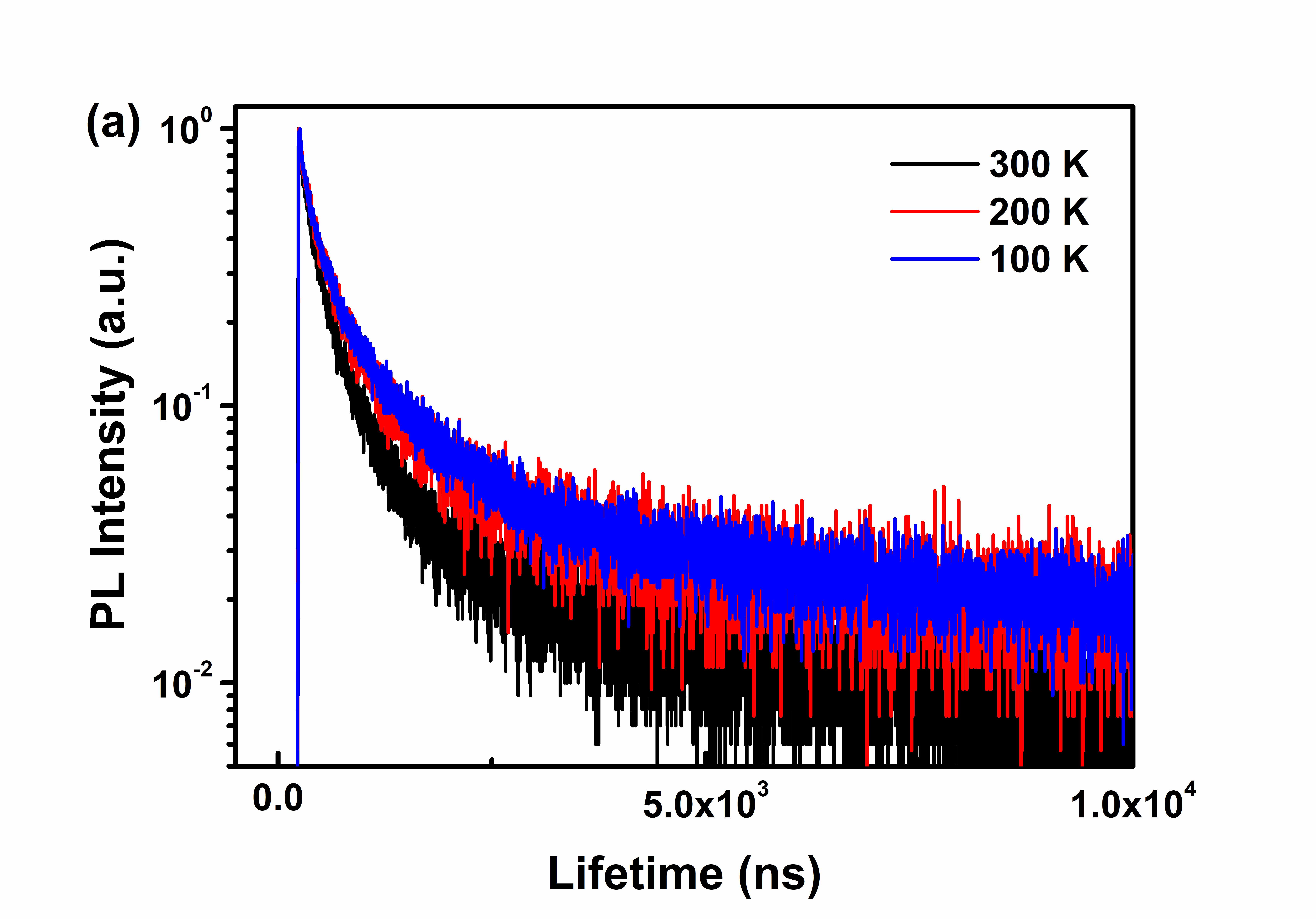

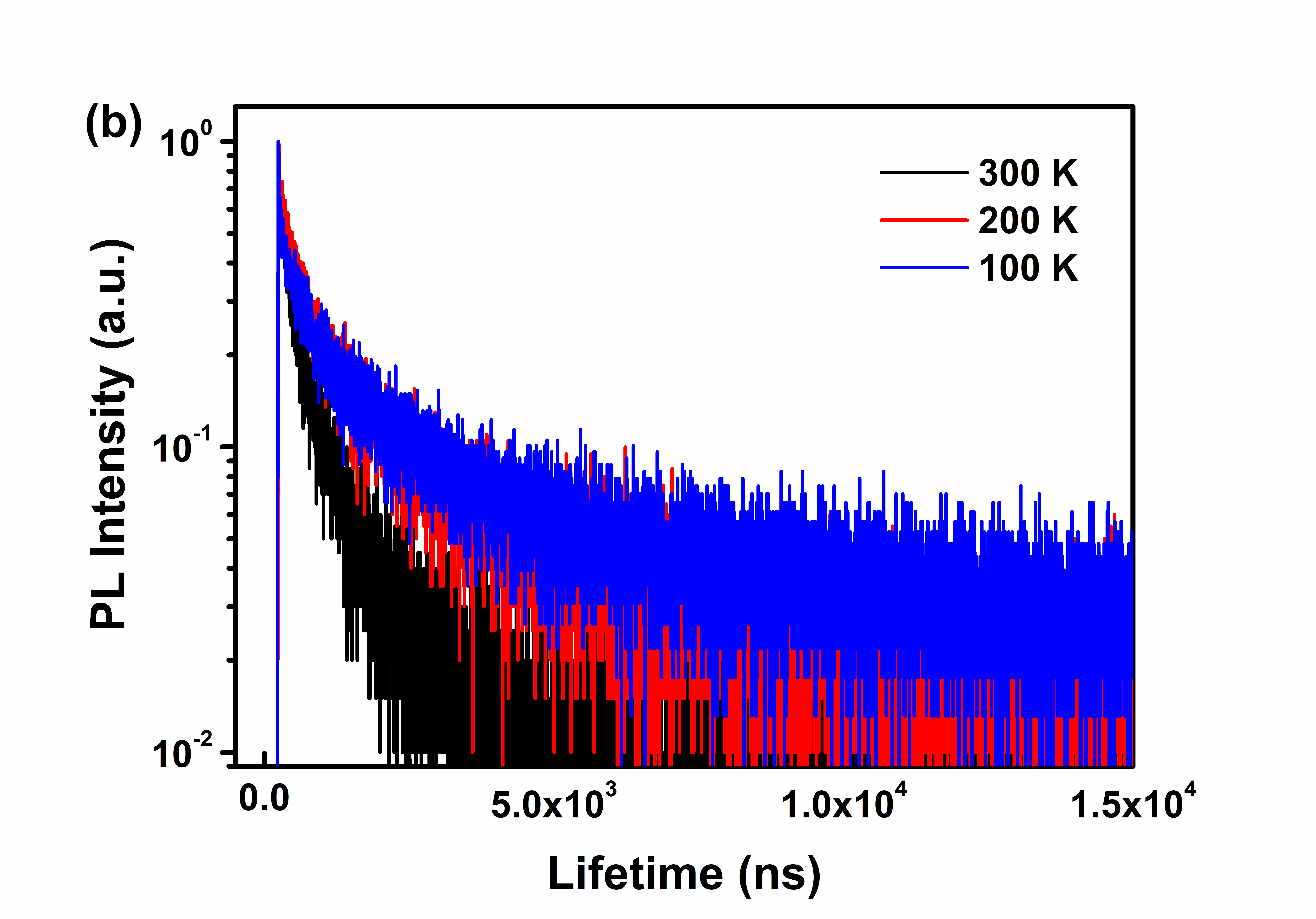
**Figure S3** Temperature-dependent transient fluorescence decays of the mixed films (a) TAPC:24*i*PBIOXD and (b) TCTA:*i*TPBIOXD.

# References

(1) Becke, A. D. (1988). Density-functional exchange-energy approximation with correct asymptotic behavior. *Phys. Rev. A* 38, 3098-3100. doi: 10.1103/PhysRevA.38.3098

(2) Lee, C., Yang, W., and Parr, R. G. (1988). Development of the Colic-Salvetti correlation-energy formula into a functional of the electron density. *Phys. Rev. B.* 37, 785-789. doi: 10.1103/PhysRevB.37.785

(3) Francl, M. M., Pietro, W. J., Hehre, W. J., Binkley, J. S., Gordon, M. S., DeFrees, D. J., et al. (1982). Self-consistent molecular orbital methods. XXIII. A polarization-type basis set for second-row elements. *J. Chem. Phys.* 77, 3654-3665. doi: 10.1063/1.444267
